# Eight tips for the implementation of the first licenced peanut allergy oral immunotherapy into clinical practice

**DOI:** 10.1186/s13223-022-00671-5

**Published:** 2022-05-09

**Authors:** Jay Portnoy, Christina E. Ciaccio, Janet Beausoleil, George Du Toit, Stanley Fineman, Stephen A. Tilles, June Zhang, Claire Lawrence, Mohamed Yassine, S Shahzad Mustafa

**Affiliations:** 1 Section of Allergy, Asthma & Immunology. Children’s Mercy Hospital, Kansas City, MO United States; 2 Section of Allergy/Immunology and Pediatric Pulmonology, The University of Chicago, Chicago, IL United States; 3 Division of Allergy and Immunology, The Children’s Hospital of Philadelphia, Philadelphia, United States; 4 Department of Women and Children’s Health (Pediatric Allergy, School of Life Course Sciences, Faculty of Life Sciences and Medicine, King’s College London, London, UK; 5 Guy’s and St Thomas’ National Health Service Foundation Trust and King’s College London National Institute for Health Research Biomedical Research Centre Translational Bioinformatics Platform, Guy’s Hospital, London, UK; 6 Children’s Allergy Service, Evelina London Children’s Hospital, Guy’s and St Thomas’ Hospital, London, UK; 7 Division of Allergy & Immunology, Emory University School of Medicine, Atlanta Allergy & Asthma, GA Atlanta, United States; 8 Aimmune Therapeutics, a Nestlé Health Science Company, 8000 Marina Blvd. Suite 300, Brisbane, CA 94005 United States; 9 Latitude Food Allergy Care, Redwood City, CA United States; 10 Acaster Lloyd Consulting Ltd, London, UK; 11 Division of Allergy, Immunology, and Rheumatology Rochester Regional Health, School of Medicine and Dentistry, University of Rochester, Rochester, United States

**Keywords:** Oral immunotherapy, Food allergy treatment, Peanut allergy, Peanut oral immunotherapy, Desensitization, Shared decision making, Allergy immunotherapy, Adherence, Implementation, Education

## Abstract

**Background:**

Shared learnings from the early use of novel therapies can aid in their optimization. The recent introduction of peanut oral immunotherapy (peanut OIT; Palforzia [Peanut (*Arachis hypogaea)* Allergen Powder-dnfp]) for peanut allergy addresses a significant unmet need but also highlights the requirement for consideration of several factors by both prescribers and patients.

**Objective:**

To provide guidance for prescribers of licenced peanut OIT to facilitate treatment delivery and improve outcomes.

**Methods:**

Clinicians with experience of licenced peanut OIT (United States n = 6, United Kingdom n = 1) participated in a series of interviews and group discussions designed to elicit tips for successful implementation.

**Results:**

Clinicians identified 8 tips that were considered the most relevant, practical, and impactful for prescribers of Peanut (Arachis hypogaea) Allergen Powder-dnfp: (1) preparing to provide treatment, (2) assessing the medical indication for treatment and (3) shared decision making, (4) staff education, (5) establishing office processes, (6) managing patient expectations and using anticipatory guidance, (7) optimising adherence and (8) maintaining flexibility throughout the treatment process. In addition, a range of supporting materials (e.g., checklists and action plans) are provided.

**Conclusion:**

The introduction of a novel therapy often requires healthcare providers to modify or adopt practices to effectively employ the treatment. The provision of guidance based upon early real-world experiences of licenced peanut OIT may help inform clinical practice and improve treatment outcomes.

**Supplementary Information:**

The online version contains supplementary material available at 10.1186/s13223-022-00671-5.

## Introduction

**Fig. 1:**
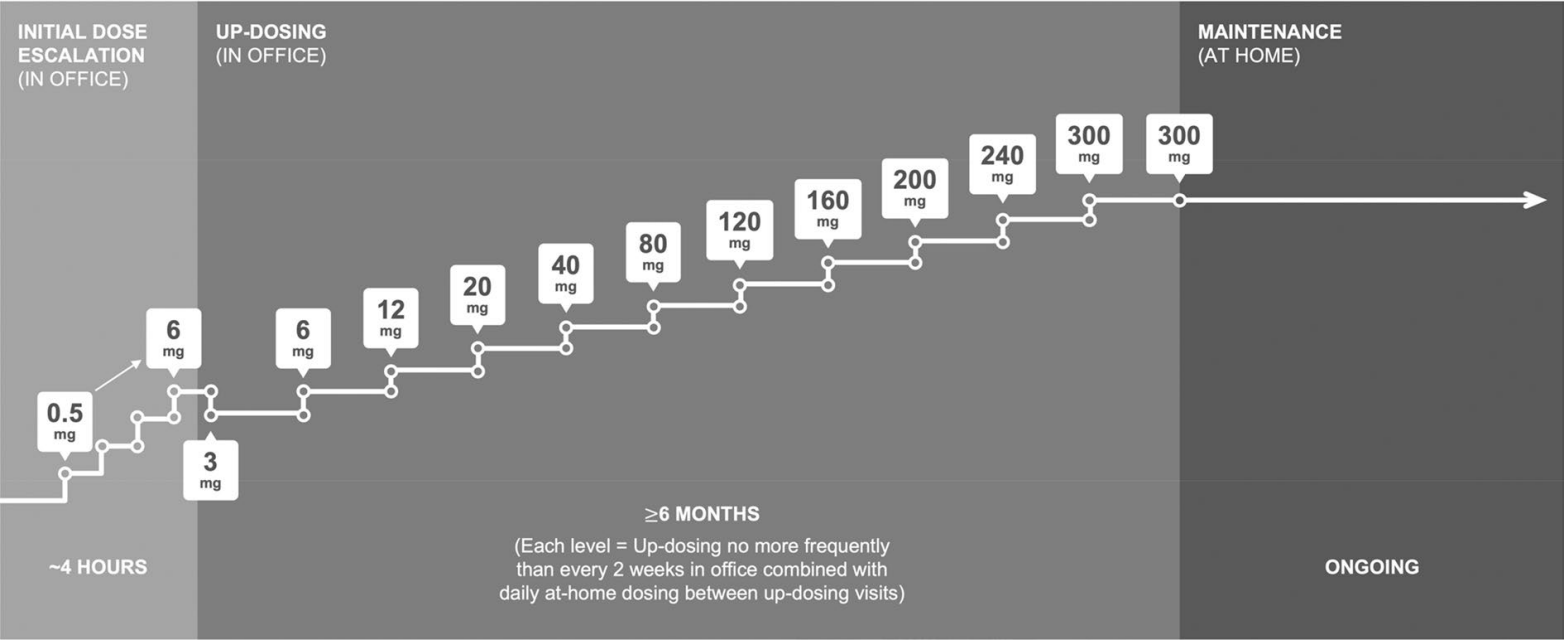
Outline of Palforzia protocol

Peanut allergy (PA) is among the most common food allergies, with a prevalence of approximately 2% in Western nations [1–5]. The increasing burden of illness is substantial and well documented [6–8]. In 2020, the first treatment for the mitigation of allergic reactions to peanut was approved by the United States (US) Food and Drug Administration (FDA) [9], followed shortly by European Commission (EC) approval [10]. Palforzia^®^ [Peanut (*Arachis hypogaea)* Allergen Powder-dnfp; defatted powder of *Arachis hypogaea* L., semen (peanuts); previously known as AR101; Aimmune Therapeutics, Brisbane, California, USA] is a licenced peanut oral immunotherapy (peanut OIT) indicated for individuals aged 4–17 years with a confirmed PA diagnosis. Although there are several published reviews summarizing how to administer food OIT in everyday practice [11, 12], most allergists have not chosen to prescribe food OIT to their patients [13]. Several reasons for low adoption rates have been identified, including the time commitment and concerns about Palforzia’s safety [14, 15]. Prescribing licenced peanut OIT involves introducing a new treatment paradigm for many prescribers (Fig. 1). Eight tips developed by early adopters and treatment pioneers are presented, highlighting the skills, logistical, and practical considerations required for implementation. Our goal is for these learnings to support implementation as licenced peanut OIT becomes more widely accessible across the US, the UK and beyond.

## Methods

**Fig. 2:**
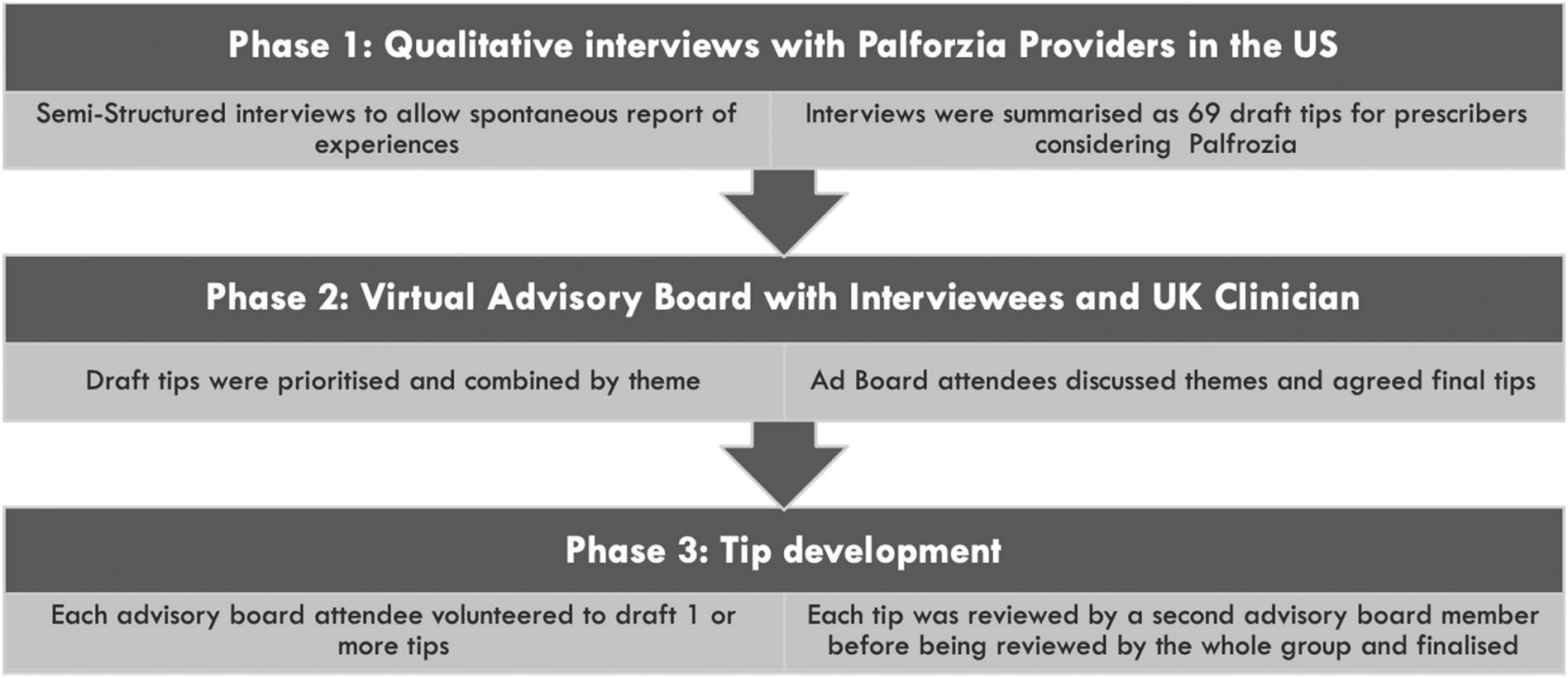
Tip generation process

A summary of the methods employed to develop the tips is presented in Fig. 2. Further details of the methods are presented in Additional file 1.

## Eight tips for the implementation of licenced peanut OIT into clinical practice

Eight tips for the implementation of licenced peanut OIT into clinical practice into the US, the UK and beyond are outlined below (Table 1).

**Table 1:** Eight Tips for the Implementation of Palforzia into clinical practice

1. Prepare for providing Palforzia
2. Assess the medical indication for treatment
3. Shared decision making is essential
4. Education is key for staff
5. Establish processes to streamline treatment
6. Manage patient expectations and use anticipatory guidance
7. Optimize adherence
8. Be flexible—it’s a marathon not a sprint

### Tip 1: prepare for providing licenced peanut OIT

**Fig.3:**
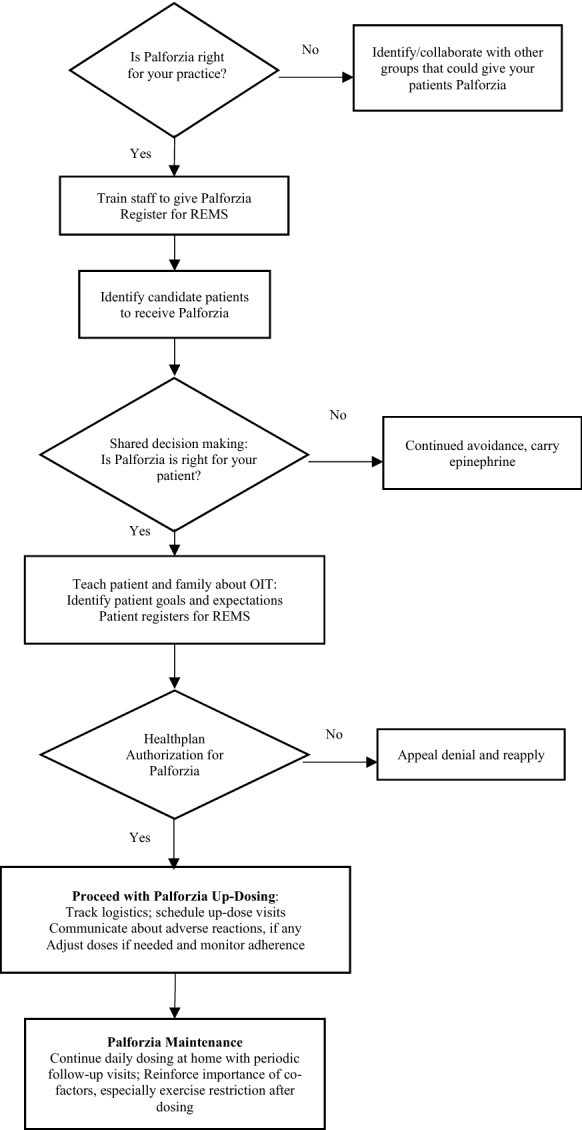
Palforzia flow-chart. Figure developed for the US. Modifications may be required for other countries

Before opting to offer licenced peanut OIT to patients, the provider should carefully consider the facilities, staffing, clinical experience, operational processes, time, and commitment required. In-office administration of OIT involves resources similar to those needed to perform an oral food challenge (OFC), including time, sufficient staff, office space, preparation to treat adverse events, and possibly hospital proximity [16]. In most practices, these perceived barriers can be overcome with proper preparation. Physicians who are already comfortable and whose office is equipped to conduct OFCs would be well suited to deliver licenced peanut OIT. A flowchart summarising the process is presented in Fig. 3.

Adequate staffing is needed to schedule patients, keep refrigerated office dose kits stocked, and order patient-specific doses. These include the initial dose escalation (IDE) card, subsequent up-dosing packs, and a final maintenance dose pack. The clinical team may involve other physicians (e.g., 24/7 call coverage), advanced practice providers to supervise the procedure, and nurses to prepare and administer doses and assess for reactions. In contrast to food OIT that is not FDA approved, with Palforzia there is no need for preparation of the allergen. Consideration of space and time is needed when assessing clinical facilities. The IDE takes three hours and is like an incremental food challenge. The 11 subsequent up-dosing visits require 60 min for monitoring.

It is also important for clinics administering licenced peanut OIT to be prepared for emergencies [17], including a health care provider with experience in recognizing and treating anaphylaxis and availability of epinephrine and other medications and equipment needed to treat severe systemic allergic reactions. Relatedly, providers and healthcare settings should prepare to follow all necessary precautions to minimize the risk of anaphylaxis with peanut OIT treatment as specified in respective licencing information. Requirements may vary depending on location. However, in the US, the FDA stipulates that the prescriber, healthcare setting and patient must enrol in a mandatory Risk Evaluation and Mitigation (REMS) program due to risk of a severe systemic allergic reaction (including anaphylaxis) [9]. The prescribers complete an attestation of their responsibilities [18], and the healthcare setting must be equipped to manage anaphylaxis and have procedures in place to assure that patients are monitored during the IDE and each up-dosing visit. Similarly, in Europe and the UK a risk-management plan (RMP) was submitted to the European Medicines Agency (EMA) when Palforzia was undergoing marketing authorisation assessment. This plan provides a detailed description of the activities and interventions designed to identify, characterise, prevent or minimise risks relating to the medicine. The Palforzia RMP has a provision that educational risk minimisation materials must be provided to the health care professionals and patients caregivers to cover important safety information [19].

### Tip 2: assess the medical indication for treatment

Confirmation of PA is essential before starting treatment. Diagnosis should be based on a clinical history suggestive of an IgE mediated reaction to peanut, accompanied by a positive peanut skin prick test and/or elevated peanut specific IgE and components [20]. Although it is not practical or necessary to perform an OFC on all patients before treatment initiation, a physician-supervised OFC should be considered if there is uncertainty regarding PA diagnosis, especially in an individual whose PA diagnosis is based on testing alone. There are multiple potential benefits to performing an OFC, including either confirming or refuting the diagnosis, establishing a threshold dose for clinical reactivity, and improved health-related quality of life, regardless of food challenge outcome [21].

Once a diagnosis is confirmed, additional patient-specific factors should be considered prior to treatment, including contraindications for OIT such as uncontrolled asthma or eosinophilic gastrointestinal disorders [9]. Relative contraindications to treatment may include certain chronic medical conditions (e.g. inflammatory bowel disease or mast cell disorders) and medications that may increase the risk of adverse events (e.g. beta blockers).

A previous history of severe systemic allergic reaction may be an important reason to pursue treatment with licenced peanut OIT. Patients with such histories were included in pivotal trials of Palforzia. However, individual practices may differ in their comfort level treating patients with a history of PA reactions involving significant hypotension or requiring intubation. Significant skin test reactivity or high levels of peanut-specific IgE are not contraindications to treatment, as these patients were included in the pivotal trials and their presence did not accurately predict success or failure with treatment [22]. Other factors to consider include details of a patient’s living situation, anxiety, or projected adherence due to busy schedules, especially a significant commitment to after-school extracurricular activities. Since the characteristics of the ideal individual/family for treatment remains unknown, careful shared decision making is critical.

### Tip 3: shared decision making is essential

As is the case with most new therapies, no one “right” answer exists as to whether one should undergo treatment. Shared decision making helps to balance risks and expected outcomes with patient preferences and values. Table 2 lists considerations that should be addressed during shared decision-making.

**Table 2:** Considerations to address before starting OIT

Review lifestyle	• Competitive sports and other extracurriculars	• May not increase heart rate for any reason including sports or other activity for 3 hours after dosing every day
	• School schedule	• Will need to be carefully monitored by caregiver to ensure dose is taken daily
	• Vacations and travel	• Up-dosing may be delayed by one or more weeks to accommodate travel or schoolwork
	• Caregiver monitoring	• Will need to be carefully monitored by caregiver for any reaction after every dose
	• Transportation	• Legally authorized caregiver should provide transportation to clinic; adolescents should not attend alone
Clarify timing of daily dose	• Early morning before school	• May require 5am wakeup
	• Immediately following school dismissal	• activity, even when weather is nice
	• Dinnertime	• Must remain awake for monitoring for 3 hours after dos
Understand goals	• Bite-safe	• Life-long daily dosing is required to remain bite safe
	• Free eat	• Free eating peanut containing foods may be possible for some but requires a monitored challenge in clinic before starting as well as careful explanation of risks
	• Remission	• No evidence currently exists that OIT induces remission, i.e. if daily dosing is stopped; protection may be lost
Review options for Mixing	• Yogurt	• Consider other food allergies
	• Ice cream	• Consider particular tastes of child
	• Guacamole	• Have multiple options available
	• Pudding	
	• Applesauce	
	• Smoothie	
Review risk factors for systemic reactions	• Exercise (or any activity that increases heart rate)	• Heart rate may not be elevated for 3 hours after each dose
	• NSAID use	• OIT is likely not possible for those on chronic NSAIDS
	• Menstruation	• Consider avoiding up-dosing appointments
	• Hot showers	• Drinking water throughout the day, particularly on up-dose days is crucial
	• Dehydration	• Each dose should be taken with a meal or substantial snack
	• Empty stomach	
Review medications that treat side effects	• Second generation H1 blocker	• Patient should have at least one of each that is tolerated
	• H2 blocker	• Family should have on hand prior to starting
		• Correct dosing should be provided

Providers should set aside time for a discussion between the patient, caregivers, and allergist that is not rushed and that may help the patient feel empowered to ask questions and engage in dialogue. For some patients and caregivers, telemedicine may be a useful platform for this encounter as it is easily accessible, allows for the involvement of additional caregivers, and can be done from the comfort of the child’s home. Depending on the patient’s age, an initial counseling visit with caregivers alone may be valuable. Providers should consider formalizing a workflow in which families carefully consider their options before signing required paperwork and prior to the pre-treatment visit.

Information offered by the provider should be easy to understand and available asynchronously. A form with frequently asked questions could be posted on the practice website and included with educational materials. Provider should consider creating or providing a video for patients to watch either in the office/clinic or on the practice website. Information should be reviewed with families regarding adverse events that may occur, especially gastrointestinal issues including throat itching, abdominal pain, vomiting, and symptoms consistent with the development of eosinophilic esophagitis, as well as the risk for anaphylaxis. A consent form should outline the above risks involved in the treatment.

### Tip 4: education is key for staff

Staff education should ideally be standardized (e.g. checklist) to ensure that every patient receives the most important information (Table 3). Training should include how to identify treatment candidates, discuss goals of treatment, the pathophysiology of OIT, proper dose administration and monitoring, management of adverse reactions, dosing adjustments for missed doses, and how to advise patients regarding fever/illness, travel, sports, and other common questions. Providers should consider creating a frequently asked questions (FAQ) handout that can be consulted and updated as new questions are identified and answered. A system for answering such questions should be established to ensure consistency.

**Table 3:** Checklist of essential components of education

Counselling information	
	Advise patient, parent, or guardian to read the FDA-approved patient labelling
	Advise patient, parent, or guardian that the patient should continue to follow a strict peanut-avoidance diet
	Advise patient, parent, or guardian that peanut OIT will not prevent allergic reactions to other foods to which they might be allergic
	Advise patient, parent, or guardian that peanut OIT may cause allergic reactions such as anaphylaxis. Teach them to recognize the signs and symptoms of anaphylaxis
	Patients should have injectable epinephrine and they should be instructed when and how to use it
	Inform the patient, parent, or guardian that the first dose of each dose level of peanut OIT must be administered in a health care setting under the supervision of a health care professional, and that after taking the dose, the patient will be monitored for signs and symptoms of an allergic reaction
	Instruct patient, parent, or guardian that patients with asthma should stop taking peanut OIT and contact their health care professional immediately if they have difficulty breathing or if their asthma gets worse
	The patient should consume the entire prepared mixture

Dosing instructions Advise patient, parent, or guardian	
	The importance of taking each dose daily to avoid loss of treatment effect
	That each dose should be taken with a meal, at approximately the same time each day, preferably in the evening
	To observe the patient for at least 60 min after administering peanut OIT for an allergic reaction
	To contact their health care professional for advice on how to resume peanut OIT if more than 2 doses are missed
	That the risk of an allergic reaction after peanut OIT may be increased in the presence of • Exercise or exposure to hot water • A medical illness such as a viral infection • Not eating for a day • Sleep deprivation • Use of nonsteroidal anti-inflammatory drugs such as aspirin or ibuprofen • Uncontrolled asthma • Alcohol consumption If one of these happens, it may be necessary temporarily to withhold or decrease the dose of peanut OIT

### Tip 5: establish processes to streamline treatment

Providers should consider establishing distinct processes for scheduling, tracking, and ordering supplies. Space and staffing should be considered when scheduling each visit, especially the IDE. Appropriately scheduling IDE visits at adequately spaced intervals can ensure there will be adequate space and staff as the number of individuals on treatment increase over time. It is imperative to allow for flexibility when scheduling visits for up-dosing, as patients may need to delay these visits due to illness or adverse reactions. Practices may find it beneficial to reserve specific days for licenced peanut OIT, or to “cohort” patients in groups.

An electronic medical record (EMR) can facilitate education, scheduling, documentation, and patient tracking. It may be useful to create OIT-specific progress note templates, visit type, and flowsheets to track dosing visits. Patients may also be given a log to record home dosing, symptoms etc., to review before each up-dose. Providers should consider implementing inventory management processes to track Office Dose Kit inventory, ensure doses are available for scheduled visits, and have semisolid food stocked in the office including choice of applesauce, yogurt, and pudding.

Other considerations such as prior authorization, insurance approval, and risk minimization programs (e.g. REMS in the US) will depend on local healthcare setting and licencing conditions.

### Tip 6: manage patient expectations and use anticipatory guidance

Patients and caregivers must understand that peanut OIT is not a cure for PA. It is important to communicate to patients that they should continue to avoid peanut and to have an epinephrine autoinjector available, even when the patient reaches the maintenance dose. Finally, it is important to tell patients that long-term (at least several years) daily dosing is required to maintain the effect of treatment.

Patients and caregivers must understand they cannot ‘adjust’ or skip doses on their own, and that all up-dosing must take place in the office. Transient gastrointestinal symptoms occur in 85% of children treated with Palforzia [22]. Anticipatory guidance regarding these likely side effects (i.e. explaining prior to initiating treatment that mild allergic side effects indicate the medicine is working) may reduce patient-related anxiety, increase adherence, and moderate office call volume. Patients should already have been trained to use an epinephrine autoinjector and they should have an anaphylaxis action plan (Additional file 2). Patient instructions regarding the treatment of adverse reactions and dose adjustments before initiating treatment can decrease the potential for after-hours calls (Additional file 3).

There should be a plan in place to manage common side effects. H1 and H2 blockers are routinely used to manage side effects of OIT [23, 24], and families should know when to rely on these interventions to help with chronic symptoms, even if mild. Practices may also consider providing families with a formal side effect action plan, similar to an emergency action plan used to manage accidental ingestions (Additional file 1). For example, step one may include the initiation of a second-generation H1 blocker followed by an H2 blocker if symptoms fail to satisfactorily resolve. Step two may include delaying up-dose visits to monthly instead of biweekly as is commonly done in clinical practice when managing patients receiving other forms of allergen immunotherapy (e.g., aeroallergen or venom immunotherapy). Other written reminders may include increasing food intake with the therapeutic dose and staying hydrated throughout the course of therapy. Despite best efforts, not all side effects will be effectively treated or prevented and a clear mechanism for communication with practice providers 24 h a day, 7 days a week should be provided. Between anticipatory guidance and access to a practice provider, most patients and families should be able to successfully navigate the OIT journey.

### Tip 7: optimize adherence

Patient adherence strategies fall into two categories—managing taste aversion and attenuating co-factors that potentiate side effects. Patients normally have an inherent aversion to the taste of peanuts [25]. In addition, Palforzia contains inactive ingredients that have a distinct taste that some patients may find unpalatable. These can lead to discontinuation, missed doses, and, with older children, hiding doses, creating a false sense of security for the family—so parental supervision is important. Providers should be creative with semisolid food, for example, chocolate flavored foods or strong flavored foods such as salsa can mask the taste. Mixing with cold foods, such as ice cream can help. The temperature of foods can not only mask the taste but can also help with decreased local symptoms such as oral itching. Drinking fluids afterwards may also decrease side effects by reducing deposition on the oral mucosa and esophagus. Due to taste aversion, side effects, and fear of peanuts, dosing can lead to a daily stressful event for the family, thus, finding ways to make it a positive and rewarding experience is essential.

It is crucial to adhere to a 3-h post-dose exercise restriction, take doses after a full meal, ensure adequate hydration, and avoid factors that increase heart rate and/or body temperature (e.g., hot showers/bath, playing physical video games). Other cofactors such as NSAIDs and alcohol also have the potential to increase adverse reactions. Parents should monitor the effects of menstruation, stress, anxiety, and sleep deprivation and should ensure asthma and allergic rhinitis are well controlled [26, 27].

### Tip 8: be flexible—it’s a marathon, not a sprint

The dosage schedule recommended for licenced peanut OIT has been extensively tested and generally is successful, however, it can be modified if needed. As with other immunotherapies, remaining flexible and personalizing the therapy to the individual is essential. Personalizing the dosing and schedule will likely lead to a lower incidence of allergic reactions and other adverse events in real-world practice compared to clinical trials. Allergists are uniquely equipped to handle this dynamic process given their long-standing experience with subcutaneous immunotherapy (SCIT).

Moving through up-dosing at a conservative pace can benefit the practice and the patient [14]. Although families may be tempted to “sprint” to maintenance dosing, current evidence does not support this as desirable or necessary, and prescribing information allows prescriber discretion, as long as up-dosing intervals are at least 2 weeks. Unlike SCIT, since patients are dosing their immunotherapy daily, up-dosing reactions should not increase if visits are extended; rather, extending the time interval between up-dosing visits allows for longer exposure to a given dose level, which may improve tolerability to the next dose level.

Although improved safety with longer up-dosing intervals has not been proven, evidence supports that immunomodulation is ongoing before the maintenance dose is achieved, even at stable lower doses [22]. Careful observation of increasing gastrointestinal side effects including mouth tingling and stomach discomfort serve as useful guides to delay up-dosing. A child who experiences a stomachache despite pretreatment with H1 and H2 blockade may benefit from a several-week “pause” where the same dose is taken at a constant or lower daily for longer than 2 weeks to allow symptoms to clear before an attempt is made to up-dose again. Additionally, a patient may simply prefer, for lifestyle accommodation, to up-dose monthly or every other month. For example, patients who are traveling long distances to see a provider may choose to space out intervals to make travel time more tolerable, or patients who have difficulty catching up after missing school may choose to up-dose only on school holidays.

## Discussion

Recent advances in the treatment of PA have led to the first FDA and EC-approved treatment for the mitigation of allergic reactions to peanut. While many children and adolescents with PA stand to benefit from licenced peanut OIT, the adoption of any new therapy requires careful thought about the skills, logistical and practical considerations required for successful implementation.

This project brought together clinicians from a range of practice settings, clinicians with and without experience of OIT for foods other than peanut, and clinicians with and without experience of Palforzia in clinical trials. All but one of the clinicians were located in the US, reflecting the landscape of licenced peanut OIT implementation at the time of the advisory board. While the inclusion of prescribers based predominantly in the US may be considered a limitation, we believe that the majority of the tips presented are relevant and of value to prescribers beyond the US and the UK. As Palforzia becomes more widely available outside of the US, it is important to consider how the tips presented in this report can be adapted to be applicable in a range of healthcare settings and locations. For example, there may be differences in requirements for prior authorization, insurance approval, and risk minimization programs. In this manuscript we do not elaborate on forms of OIT that are not yet approved by the FDA or EC. For example, some allergists may elect to offer licenced OIT for PA alongside tree nuts, egg, and milk for patients with those allergies. Our intent is not to neglect these other OIT practices, but rather to complement them with information specific to licenced peanut OIT while referring interested readers to prior reviews for descriptions of un-approved forms of OIT [11, 12].

We hope that these tips will assist a wide range of potential prescribers to offer licenced peanut OIT. In addition to benefitting patients and families who have for so long endured the burden of untreated food allergies, providing successful OIT treatment in a clinical practice can be a source of profound professional satisfaction, and it is our hope that this manuscript will help facilitate the safe addition of Palforzia into routine allergy practice.

## Supplementary Information

**Additional file 1**: Methods

**Additional file 2**: Example food allergy action plan (source: Children’s Mercy Hospital)

**Additional file 3**: Palforzia dosing instructions to send home with patients

## Data Availability

Data sharing is not applicable to this article as no datasets were generated or analysed during the current study.

## Abbreviations

EMR: Electronic medical record
FDA: Food and Drug Administration
IDE: Initial dose escalation
OFC: Oral food challenge
OIT: Oral immunotherapy
PA: Peanut allergy
REMS: Risk Evaluation and Mitigation Strategy
SCIT: Subcutaneous immunotherapy
US: United States
UK: United Kingdom
