# Supplementary material for: Eight tips for the implementation of the first licenced peanut allergy oral immunotherapy into clinical practice
Source: Allergy Asthma Clin Immunol. 2022 May 9;18:37. doi: 10.1186/s13223-022-00671-5 (PMC9088027; doi:10.1186/s13223-022-00671-5)
Supplement: Supplementary file 1 — Additional file 1: Methods [file 13223_2022_671_MOESM1_ESM.docx]

**Additional file 1:** Methods

## Project approach

A heterogeneous group of allergists with experience treating peanut-allergic patients with Palforzia contributed to this project. Clinical and administrative tips to support implementation of Palforzia into practice were developed through three phases: (1) an initial tip elicitation exercise, (2) an advisory panel meeting to select and develop tips, (3) drafting of tips by contributors.

| **Table 1**. Allergist contributors to Tips | | | | | |
| --- | --- | --- | --- | --- | --- |
| **Contributor** | **Practice setting** | **Food OIT experience independent from Palforzia** | **Palforzia clinical trial investigator** | **Palforzia prescribing experience since FDA-approval †** | **Geographic location** |
| SM | Employed by large multi-specialty health system | no | no | yes | Northeast US |
| JB | Single specialty private practice | no | no | yes | Northeast US |
| SF | Single specialty private practice | no | yes | yes | Southeast US |
| GDT | Single specialty private practice | no | yes | no‡ | London, UK |
| JZ | Single specialty private practice | yes | no | yes | West Coast US |
| CC | Academic practice | yes | yes | yes | Midwest US |
| JP | Academic practice | yes | yes | yes | Midwest US |
| † As of October 2020  ‡ Practices in UK (Palforzia was not yet available commercially | | | | | |

## Project contributors

Each contributor has experience managing patients treated with Palforzia (Table 1) in clinic and/or as part of research studies.

## Tip development process

Qualitative semi-structured interviews to elicit tips were conducted by an independent research agency. An interview guide was employed which encouraged contributors to spontaneously suggest tips that might be useful for allergists without OIT experience who are planning to incorporate Palforzia into their practices. A total of 69 tips were generated by the 6 US contributors. The advisory panel meeting focused on narrowing down the tips, after which all 7 contributors agreed on the final list of 8 tips.

The advisory panel meeting was attended virtually by the six US-based allergist contributors and a UK-based allergist who was not involved in the qualitative interview phase. Contributors discussed tips they considered to be the most relevant and potentially impactful. All tips were proposed and developed solely by the practicing allergist contributors based on their Palforzia prescribing experience. Following the advisory board, each contributor agreed to draft one or more of the tips. Each tip was then reviewed by a second advisory board member before being reviewed and finalised by all contributors.
