# Supplementary material for: Eight tips for the implementation of the first licenced peanut allergy oral immunotherapy into clinical practice
Source: Allergy Asthma Clin Immunol. 2022 May 9;18:37. doi: 10.1186/s13223-022-00671-5 (PMC9088027; doi:10.1186/s13223-022-00671-5)
Supplement: Supplementary file 2 — Additional file 2: Example food allergy action plan (source: Children’s Mercy Hospital) [file 13223_2022_671_MOESM2_ESM.docx]

**Additional file 2. Example food allergy action plan (source: Children’s Mercy Hospital)**

*This information is shown for illustration purposes only and does not represent the views of Aimmune Therapeutics.*

**ALLERGY TO: _**

| **Reaction** | **System** | **Symptoms** | **Do the following** |
| --- | --- | --- | --- |
| **No Reaction** |  | - Exposure to allergen but no symptoms | Observe |
| **Mild Reaction** | Nose  Mouth  Skin  Abdomen | - Runny nose, sneezing - Itching, tingling or swelling of lips, tongue or mouth - Hives, itchy rash, swelling of face or extremities - Nausea, cramps, vomiting, diarrhea | Give antihistamine and  Observe  **Note**: Severity of symptoms can change quickly. |
|  | **Reaction involves 2 or more systems or it involves:** | |  |
| **Moderate to Severe Reaction** | Throat  Lung  Heart | - Tightening of throat, hoarseness, cough - Labored breathing, wheezing - Fast pulse, fainting, blue skin | Use Epinephrine* and then  give antihistamine  Call 911 if 2^nd^ dose is used |

*May repeat epinephrine dose in 10 minutes if symptoms have not resolved.

**Medications:**

**Antihistamine**: Benadryl (diphenhydramine) _

**Epinephrine**: Inject into the muscle of the thigh: (_)

Epinephrine autoinjector 0.3mg (_)

Epinephrine autoinjector 0.15mg ( )

**Emergency Contacts:**

- Name: ________________________________________________________

Relationship: __________________

Home Phone Number: (_______) _______-__________

Work Phone Number: (_______) _______-___________

- Name: ________________________________________________________

Relationship: __________________

Home Phone Number: (_______) _______-__________

Work Phone Number: (_______) _______-___________

**THIS PLAN SHOULD BE FOLLOWED EVEN IF PARENT/GUARDIAN CANNOT BE REACHED**

|  |  |  |  |  |
| --- | --- | --- | --- | --- |
| Signature/Relationship of Person Responsible for Healthcare Decisions |  | Printed Name |  | Date |

|  |  |  |  |  |
| --- | --- | --- | --- | --- |
| Signature of Provider |  | Printed Name |  | Date |

**Other Anaphylaxis Action Plans:**

American Academy of Allergy, Asthma & Immunology: <https://www.aaaai.org/aaaai/media/medialibrary/pdf%20documents/libraries/anaphylaxis-emergency-action-plan.pdf>

Australasian Society of Clinical Immunology and Allergy: <https://www.allergy.org.au/images/stories/anaphylaxis/2020/ASCIA_Action_Plan_Anaphylaxis_EpiPen_Red_2020.pdf>

Food Allergy Research & Education: <https://www.foodallergy.org/living-food-allergies/food-allergy-essentials/food-allergy-anaphylaxis-emergency-care-plan>
