# Supplementary material for: Eight tips for the implementation of the first licenced peanut allergy oral immunotherapy into clinical practice
Source: Allergy Asthma Clin Immunol. 2022 May 9;18:37. doi: 10.1186/s13223-022-00671-5 (PMC9088027; doi:10.1186/s13223-022-00671-5)
Supplement: Supplementary file 3 — Additional file 3: Palforzia dosing instructions to send home with patients [file 13223_2022_671_MOESM3_ESM.docx]

**Additional file 3. Palforzia dosing instructions to send home with patients**

*This information is shown for illustration purposes only and does not represent the views of Aimmune Therapeutics.*

Home Instructions for Peanut Oral Immunotherapy with Palforzia

Your current dose is ( ) mg. This dose should be mixed with a semi-solid unheated food and given once daily.

Dosing Schedule:

- Doses of Palforzia ideally should be taken **21-24 hours apart**.
- You can miss doses up to 2 consecutive days and then resume the previous dose. If you miss doses for more than 2 days, consult your healthcare provider before taking the next dose.
- Let us know if you have a respiratory infection or if you are having problems with asthma when it is time for you next dose increase. It may be necessary to postpone your appointment since respiratory infections and asthma can increase the likelihood of a reaction.

Precautions:

- Remember to **avoid exercise for at least 3 hours after each dose**.
- If your mouth has any type of injury (eg: cracked lips, loose teeth, mouth sores) try to protect that area from exposure to the Palforzia. You can try using Vaseline or a straw to avoid the lips or other affected areas. Rinse your mouth after taking the dose.
- Let us know if you have **any reaction to Palforzia**, no matter how small. It may be necessary to adjust or delay increasing your dose.

Treatment of Reactions:

- You should have an anaphylaxis plan and Epinephrine available when taking Palforzia.
- If you have an allergic reaction after a dose of Palforzia, treat it the way you would treat an accidental exposure to peanut. You should follow your anaphylaxis plan.
- If the reaction consists only of one or a few hives, you can hold off on giving antihistamine for the first hour to see if the reaction progresses. If the hives increase you should give an antihistamine.
- If you experience sneezing, wheezing, cough, shortness of breath, swelling of the mouth or throat, change in voice quality, more than one episode of vomiting or sudden quietness, **give the Epinephrine** and seek medical attention.

Call us at (Phone number) if you need to use epinephrine to get instructions on future dosing.
